# Surgeon-General’s Report, U.S.A.

**Published:** 1871-12

**Authors:** 


Circular No. 3, War Department, Surgeon-General's Office, Washington, August 17, 1871. A report of Surgical Cases treated in the Army of the United States, from 1865 to 1871, by George A. Otis, Assistant Surgeon, U. S. A.

The first eighty-seven pages of this report are devoted to the consideration of gunshot wounds. The two hundred and ninety-seven reports given furnish more or less complete data respecting three hundred and eighty-seven patients with gunshot wounds. Incised and punctured, lacerated and contused wounds are next considered. Under the heading of "Simple and Compound Fractures and Luxations," a large number of cases are detailed. The remaining cases considered come under the following headings: Arrow Wounds; Poisoned Wounds; Burns; Scalds, and Frost-bites; Amputations in the Upper Extremities; Excisions; Ligations; Various Operations on the Eye, Ear, Mouth, Chest, Abdomen, Genito-Urinary Organs, etc.

The chapter on surgical operations shows a very favorable exhibit of the results of the major operations. In a summary at the close of the report the writer says in regard to them: "Those at the shoulder and knee-joint had a larger measure of success than usually rewards the efforts of surgeons, and the small ratio of mortality in the thigh operations is exceptional. Two of the amputations in the thigh were of special interest as performed for the consequences of gun-shot fractures of the femur, inflicted five and seven years previously. The pathological specimens furnished from these cases, very imperfectly represented by the wood cuts, are very instructive. There was a successful case of amputation of the fore-arms and of the legs in one patient, and one of the two exarticulations of the hip had a successful issue."

"The twenty-seven reports of ligations of the larger arterial trunks include one in which the common carotid was successfully tied for secondary hæmorrhage following a gun-shot wound of the face and neck, and supposed to involve the external carotid near the origin. That there should not have been recurrent hæmorrhage from the distal orifice in the vessel is surprising. A compulsory ligation of the aorta for rupture of an aneurism of the common iliac is reported from one of the freedmen hospitals—one of three ligations of the iliac proved successful. Three of the four ligations of the femoral resulted happily. The fatal case was one in which Anel's operation was performed, neither experience nor theory having convinced the operator that tying the femoral in its middle third would not preclude the fatal consequences of recurrent haemorrhage from a wound of the popliteal. It is almost incredible, but there are still many surgeons who think it unnecessary to place two ligations on wounded arteries, but are satisfied in securing the proximal extremity or in tying the main trunk at a distance. The reports indicate that acupressure was not employed to any extent." An interesting case of brachial aneurism successfully treated by compression is recorded in page 155.

The volume is illustrated by numerous wood cuts and by three fine lithographs, the first illustrating Otis' successful rè-amputation at the hip-joint, the second showing Gibson's successful excision at the hip. Considered as a whole, the circular is a very valuable one and presents a large amount of matter, particularly important to the medical officers of the army, for whose more especial information and instruction it is published. At the same time the facts and ideas brought forward will prove of equal value and assistance to the general surgeon.

